# Elastography Enhances the Diagnostic Performance of Conventional Ultrasonography in Differentiating Benign from Malignant Superficial Lymphadenopathies

**DOI:** 10.3390/cancers17091480

**Published:** 2025-04-28

**Authors:** Novella Pugliese, Marco Picardi, Claudia Giordano, Annamaria Vincenzi, Rosaria Cappiello, Massimo Mascolo, Fabrizio Pane

**Affiliations:** 1 Department of Clinical Medicine and Surgery, Hematology Section, University of Naples “Federico II”, Via Sergio Pansini, 5, 80131 Naples, Italy; marco.picardi@unina.it (M.P.); claudia.giordano@unina.it (C.G.); annamaria.vincenzi@unina.it (A.V.); fabpane@unina.it (F.P.); 2 Department of Advanced Biomedical Sciences, Pathology Section, University of Naples “Federico II”, Via Sergio Pansini, 5, 80131 Naples, Italy; rosaria.cappiello@unina.it (R.C.); massimo.mascolo@unina.it (M.M.)

**Keywords:** elastography, ultrasonography, lymph node, lymphoma, strain ratio, diagnostic accuracy

## Abstract

Background/Objectives: Lymph node (LN) evaluation is critical in diagnosing, staging, and managing various diseases, particularly lymphoma and metastatic cancer. Although conventional ultrasound (US) is widely used for this purpose, its limitations in reliably differentiating between benign and malignant LNs persist. Ultrasound elastography (US-E), which evaluates tissue stiffness, has emerged as a promising adjunct to improve diagnostic accuracy. This study aims to evaluate the diagnostic performance of conventional US, power Doppler US, and strain elastography (SE) in distinguishing malignant from benign superficial lymph nodes. Methods: In this prospective study, 214 consecutive patients referred for US of enlarged LNs were enrolled. Conventional B-mode US, power Doppler, and SE were performed, and the strain ratio (SR) was calculated as a measure of LN stiffness. Histopathological examination was used as the reference standard. Diagnostic accuracy was assessed using receiver operating characteristic (ROC) analysis, and multivariable logistic regression models were applied to determine the independent predictive role of SR. Results: Among the 214 LNs (one for each patient), 74 (34.6%) were benign and 140 (65.4%) were malignant. The SR showed a significant association with malignancy (*p* < 0.001). For hematological malignancies, SR demonstrated high sensitivity (79–85%) and specificity (81–96%), with an overall area under the curve (AUC) of 0.91. Multivariable analysis confirmed that SR was an independent predictor of malignancy (continuous and dichotomous), with a 14% gain in predictive accuracy when treated as a continuous variable (*p* < 0.0001). Conclusions: US-E, particularly SR, is a valuable tool in the differentiation of benign and malignant superficial LNs. SR provides significant diagnostic value, especially in hematological neoplasms like Hodgkin lymphoma, and can serve as an independent predictor of malignancy. This technique, when used in combination with conventional US features, offers enhanced diagnostic performance for LN evaluation.

## Simple Summary

This study evaluates the use of ultrasound elastography (US-E) to differentiate between benign and malignant superficial lymph nodes. The results show that the strain ratio, a measure of tissue stiffness, can significantly improve the accuracy of lymph node assessments, especially in diagnosing lymphoma. These findings highlight the potential of US-E to serve as a valuable, non-invasive tool in clinical practice, potentially guiding the selection of targets for biopsy, improving diagnostic precision.

## 1. Introduction

Lymph node (LN) evaluation plays a central role in the diagnosis, staging, and management of a variety of diseases, particularly lymphoma and metastatic cancer [1,2].

Ultrasound (US), a commonly used and conventional imaging technique, has long been employed for this purpose due to its safety, accessibility, and relatively low cost [3,4].

The introduction of new-generation US devices in clinical practice has enhanced the recognition of the ultrasonographic characteristics of both benign and neoplastic LNs [5,6,7] and the addition of power Doppler evaluation, which allows for the study of neoangiogenesis in LN tissue [8,9,10,11,12], has further improved the diagnostic performance of US.

Although established ultrasonographic criteria exist for differentiating benign from malignant LN, the US is unable to consistently achieve high sensitivity and specificity simultaneously [2,13,14].

In recent years, US elastography (US-E), a novel imaging technique that assesses stiffness or elasticity properties of biological tissues, has emerged as a promising tool in the characterization of malignant from benign lesions in various organs, including the breast [15], thyroid [16], prostate [17], liver [18], and lymph nodes [8,19,20,21], and has only recently been available on commercial clinical US systems.

It provides real-time information about tissue stiffness based on the principle that malignant tissues tend to be stiffer than benign ones [2].

Over time, various static and dynamic methods of US-E emerged. However, real-time techniques available for clinical use today are strain elastography (SE) [19] and shear wave elastography (SWE), which includes acoustic radiation force impulse (ARFI) based techniques and transient elastography (TE) [22].

SE works by applying pressure through the transducer or with physiological patient motion (breathing and heartbeat) [23]. It provides a natural extension of the B-mode imaging examination as both images can be displayed simultaneously. The most useful frames for interpreting strain images with good signal-to-noise ratio are those with a constant rate of displacement, that is, during the time of downward or upward movement of the transducer. The result is visualized within a box, where it can be represented in different chromatic scales. The signal is then represented on the screen in a color-coded manner: blue for harder tissues, and red/green for those that are softer [23]. LN elastographic analysis can be reported using a simplified 5 [24] or 4-point scale elasto-score (ES) [19]. According the four pattern system, scores 1 (soft LN that is predominantly red, with <10% of the area colored as blue) and 2 (moderately soft LN that is predominantly red and green, with 10–50% of the area shown as blue) are accepted as most likely benign, while scores 3 (moderately stiff LN that is predominantly blue and green, with 50–90% of the area shown as blue) and 4 (stiff LN that is predominantly >90% blue) are regarded as most likely malignant [19,25,26]. In an alternative approach, two regions of interest (ROIs) are defined: one over the target lesion and the other over an adjacent reference area, such as normal muscle or subcutaneous fat. The strain ratio (SR) is then automatically computed by the US machine such that values >1 indicate that the target lesion has a lower strain than the reference tissue, indicating higher stiffness. The likelihood of malignancy increases as the SR increases [19].

SE is the most frequently described method in LN assessment, as it is widely available on most commercial systems and supported by substantial evidence from numerous individual studies and two meta-analyses. These studies report sensitivities of 74–76% for ES and 83–88% for SR, along with specificities of 88% and 91%, respectively [27,28]. More recently, SWE has also been extensively evaluated, with meta-analyses further confirming its clinical value [29,30].

Furthermore, promising findings indicate that US-E provides improved diagnostic accuracy over B-mode ultrasound, and it has demonstrated potential as an adjunctive technique in LN assessment [21,31]

The aim of this prospective study was to evaluate the diagnostic performance of US features, including conventional B-mode US, power Doppler US, and SE, in distinguishing malignant from benign superficial LN. The primary endpoint was to evaluate the diagnostic accuracy of LN stiffness, as measured by strain ratio (SR), in predicting malignancy. The secondary endpoints included the following: (1) comparing the diagnostic accuracy of SR with other sonographic and elastographic features, such as LN shape, size, echogenicity, hilus presence, vascularity, and elastography (ES); (2) evaluating the optimal SR cut-off values and their accuracy in differentiating between various types of malignancies; and (3) determining the independent predictive role of SR in diagnosing LN malignancy.

## 2. Materials and Methods

### 2.1. Patients

From January 2021 to December 2023, 214 consecutive patients who were referred for ultrasonography (US) of enlarged LN were enrolled.

The study protocol was approved by the institutional review board. Before enrollment, each patient gave written informed consent, according to the requirements of the Helsinki Declaration.

In this prospective study, patients were required to meet the following eligibility criteria: (a) age ≥ 18 years, (b) LN enlargement clinically suspected of lymphoma, and (c) indication to perform nodal biopsy. Patients affected by Epstein–Barr virus, cytomegalovirus, herpes simplex virus, rubella, toxoplasma, or tuberculosis infection were excluded.

### 2.2. Sonographic Examination

All patients underwent conventional US examination, involving B mode US and power Doppler US. Conventional US features include shape, long axis measure, echogenicity, lymph node hilum, and vascularity. The shape was categorized as round (long axis/short axis < 2) or oval, size as long axis > or <1.5 cm, echogenicity as predominantly hypoechoic or not, and hilum as present or absent. Vascularization was defined as abnormal in the case of intranodal arterial vessels with a high resistive index value (≥0.6) or anarchic morphology.

LNs were considered suspicious for malignancy if at least three of the following US features were present: (a) long axis greater than or equal to 1.5 cm, (b) round shape, (c) hilus absent, (d) hypoechoic parenchyma, and (e) hypervascularization, (i.e., intranodal arterial vessels with high resistive index value [≥0.6]).

US images were obtained by 2 hematologists (M.P., N.P. with more than 10 years of experience with the conventional US for superficial and deep-seated lymph node assessment) trained in the diagnostic US who used a scanner (iU22; Philips Healthcare, Bothell, Wash) equipped with tissue harmonic compound technology (SonoCT; Philips), power Doppler sonography, and 9–3-MHz (9–3 linear; Philips) and 18-4-MHz (18-4 linear; Philips) broadband probes, as reported in detail elsewhere [5,10,12]. Each complete examination required an average of 40 min (range, 30–60 min). When discrepancy occurred, an agreement was reached after discussion.

### 2.3. Elastogram Evaluation

After gray-scale and power Doppler US, a new set of radiofrequency echo data for US-E for each LN was acquired, with the same digital sonography scanner (iU22; Philips Healthcare, Bothell, WA, USA), also equipped with the Real-time Tissue Elastography (RTE). The transducer was an electronic linear probe with 18-4 MHz. The eL18-4 transducer supported a complete elastography solution, with highly sensitive strain imaging to use to rapidly assess relative lymph node tissue stiffness values across a variety of applications, utilizing a unique pulsing scheme to generate and detect the propagation speed of shear waves, providing an absolute measure of tissue stiffness. The ability to combine this method of elastography delivered excellent imaging performance.

The result is visualized within a box, where it can be represented in different chromatic scales. Software provided by the manufacturer (QLAB 4.6.7^®^, Philips, Andover, MA, USA) furnishes a numerical value that represents the stiffness of the tissue being examined (SR). When the elastography image had stabilized (as confirmed by an indicator on the side of the screen), we recorded three 20-frame loops of RTE. The size and position of the elastographic box were selected to include the target LN including surrounding tissue in almost the same proportion while avoiding tissues (bone, blood vessels) that might disturb the appropriate analysis of the relative hardness of the target lymph node.

Using the software provided by the manufacturer, we calculated the SR (expressed as an absolute number) for each frame, obtained from the ratio of a first ROI-1 deformation value to a second ROI-2 deformation value; ROI-1 was placed over the target LN while ROI-2 included the surrounding muscles [19]. To avoid stress decay over the examination depth, the ROI for the muscle tissue was placed at a depth similar to the depth of the analyzed LN.

For each loop of 20 images, we calculated the mean SR, and the mean of the three loops was calculated for each patient.

LN elasticity was also reported as ES according to the four pattern system as previously described [19,25,26]. Operator agreement procedures were specifically applied to the assessment of the ES. In instances of an initial discrepancy between the two operators, the elastographic loops were jointly reviewed, and a final ES classification was established by consensus.

### 2.4. Reference Standard

The reference standard for defining the US technique’s diagnostic accuracy was a histopathologic examination.

The status of LN seen as positive at US and/or elastographic images and clinically suspected (according to the features described elsewhere) [5,13,14], was systematically checked with US-guided core needle biopsy [5,9,10].

After the US images were acquired, an LN biopsy was carried out at the same site used for the sonographic examination. Biopsies were carried out percutaneously with ultrasound guidance by the same operator (as described elsewhere [5,9,10,12]). All the histologic specimens were adequate for evaluation. They were examined by a pathologist who was unaware of the elastographic findings. Overall, biopsies were categorized as positive for malignancy (samples containing an adequate number of cells with morphologic atypia and evidence of monoclonality), and negative for malignancy (samples containing an adequate number of cells with no evidence of malignancy). Patients classified as having histologic results negative for malignancy underwent strict follow-up by clinicians for the following months, in order to discover a malignant disease undetected at first biopsy.

In this study, we evaluated the diagnostic value of the following 7 sonographic features to differentiate malignant and benign superficial LN. Sonographic features included shape (1), long axis size (2), echogenicity (3), hilum (4), vascularity (5), ES (6), and SR (7). The pathological result of the core needle biopsy of LN was the golden standard.

### 2.5. Statistical Analysis

Numerical variables were recorded and analyzed as median [min-max] while categorical variables were expressed as frequencies and percentages. Comparisons between groups were based on the Mann–Whitney or Chi-square tests. The predictive accuracy of SR was evaluated using receiver operating characteristic (ROC) analysis and quantified in terms of area under the curve (AUC) and corresponding 95% confidence interval (95% C.I.). The independent role of SR in predicting the diagnosis of malignancy was assessed using multivariable logistic regression models both as continuous and dichotomous variables; a bootstrap approach, based on 3000 bootstrap replications, was used to compare the percentage change in predictive accuracy (in terms of AUC) between nested logistic models. Statistical analyses and modeling were performed with R statistical computing software v. 3.6.0 (R Foundation for Statistical Computing, Vienna, Austria). *p*-values < 0.05 were considered statistically significant.

## 3. Results

### 3.1. Demographic Characteristics of Enrolled Patients and Reference Standard Findings

We analyzed 214 LNs from 214 patients, among them 108 (50.5%) were female and 106 (49.5%) were male, with a median age at US evaluation of 54.5 years (range 25–89 years).

LN stations examined were neck (*n* = 102), of which 36 were in the VB region [32], axillary (*n* = 59), and inguinal (*n* = 53) stations, respectively.

Among 214 LNs evaluated, 74 (34.6%) were benign and 140 (65.4%) were positive for malignancy at histological examination. In the group of malignant LNs, the diagnosis includes Hodgkin Lymphoma (HL) (*n* = 48), diffuse large B cell lymphoma (DLBCL) (*n* = 28), follicular lymphoma (FL) (*n* = 22), metastasis of solid tumor (*n* = 18), chronic lymphocytic leukemia/small lymphocytic lymphoma (CLL/SLL) (*n* = 11), acute lymphoblastic lymphoma (ALL) (*n* = 4), mantle cell lymphoma (*n* = 3), nodal marginal zone lymphoma (MZL) (*n* = 3), and nodal T-cell non-Hodgkin lymphoma (T-NHL) (*n* = 3).

### 3.2. Conventional Power Doppler US Finding and Diagnostic Accuracy

The US characteristics of the examined LNs and the diagnostic accuracy of each of the features tested are detailed in Table 1.

Long axes larger than 1.5 cm were observed in 90.5% (67/74) of the benign and 92.1% (129/140) of the malignant LNs, thus explaining a low sensitivity (0.39) and moderate specificity (0.66) for this criterion.

Slender-shaped nodes are more often benign (31/74, 41.9% benign vs. 21/140, 15% malignant LNs), with a specificity of 0.73, while round-shaped nodes are more common in malignant cases (43/74, 58.1% benign vs. 119/140, 85%), making them more predictive of malignancy, with a sensitivity of 0.6.

The presence of a hilum is more typically observed in benign LNs (14/74, 18.9% of benign nodes vs. 7/140, 5% of malignant LN). Conversely, the absence of the hilum is more common in malignant nodes (60/74, 81.1% of benign vs. 133/140, 95% of malignant LNs), providing a sensitivity of 0.67 and specificity of 0.69. This suggests that the presence of a hilum is somewhat indicative of benignity, while the absence of the hilum is more strongly associated with malignancy, although the moderate sensitivity and specificity indicate that it is not a perfect diagnostic criterion.

With respect to echogenicity, 95.7% (134/140) of malignant LNs exhibited hypoechoic features, compared to 83.8% (62/74) of benign LNS. On the other hand, 16.2% (12/74) of benign nodes showed hypertrophic cortical echogenicity, whereas only 4.3% (6/140) of malignant nodes displayed this feature. These findings result in a sensitivity of 0.67 and a specificity of 0.68, indicating that while hypoechoic features are more common in malignant nodes, the ability of this criterion to accurately distinguish between benign and malignant nodes is moderate.

A total of 36 of 74 (48.6%) benign nodes showed normal vascularization, while only 11.4% (16/140) of malignant nodes exhibited this pattern. On the other hand, 51.4% (38/74) of benign nodes also showed signs of hypervascularization, but this feature was much more common in malignant nodes, with 88.6% (124/140) of them exhibiting abnormal blood flow. This difference in vascular patterns results in a sensitivity of 0.69 and a specificity of 0.77, indicating that vascularization is a relatively strong predictor for malignancy, with a good balance of true positives and true negatives.

### 3.3. Elastography Diagnostic Accuracy Results

Distribution and results in terms of sensitivity and specificity of ES features in discriminating between malignant and benign lymph nodes are reported in Table 1.

According to the four pattern system, ES 1 was observed in both 1 (1.4%) benign and 1 (0.7%) malignant LN, ES 2 was found in 23 (31.1%) benign and 10 (7.1%) malignant nodes, pattern ES 3 in 33 (44.6%) benign and 52 (37.1%) malignant nodes, while ES 4 was seen in 17 (23%) benign and 77 (55%) malignant nodes. This system yielded a sensitivity of 0.47 and specificity of 0.82, indicating that while ES 4 is more commonly associated with malignancy, the overall diagnostic accuracy of the elastography patterns provides valuable information for distinguishing benign from malignant LNs.

**Figure 1:**
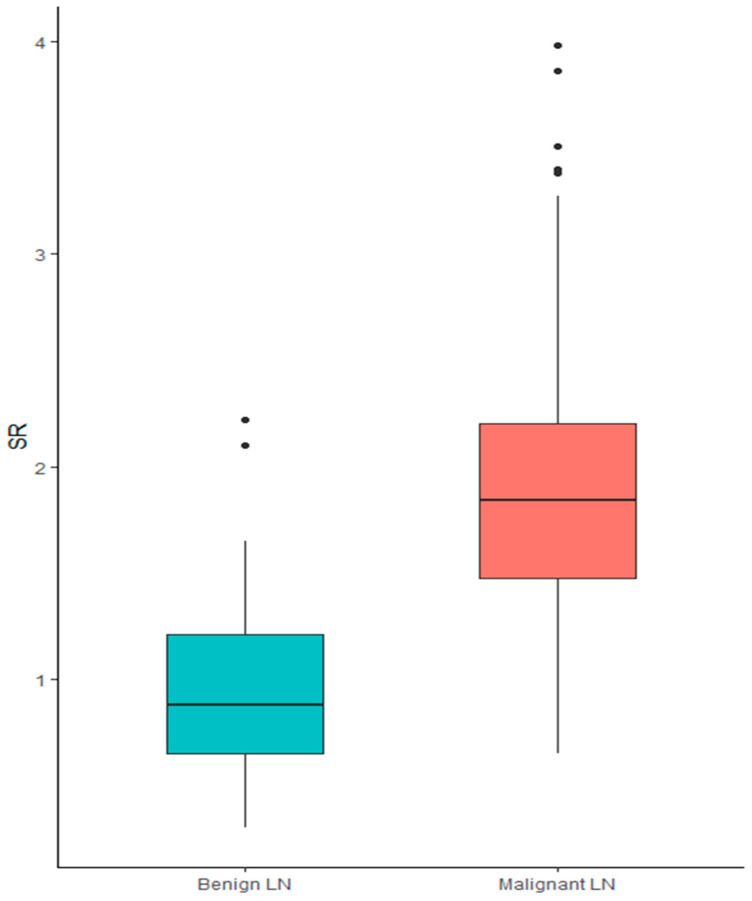
Box plot of SR in the diagnosis of benign vs. malignant lymph node.

SR showed a significant association with histological diagnosis. In particular, when treated as a continuous variable, higher SR was associated with a diagnosis of malignancy (median SR for benign LNs 0.88 [0.3–2.2] vs. 1.84 [0.65–3.98] for malignant nodes; *p* < 0.001), showing a sensitivity of 0.89 and specificity 0.78, Figure 1.

**Figure 2:**
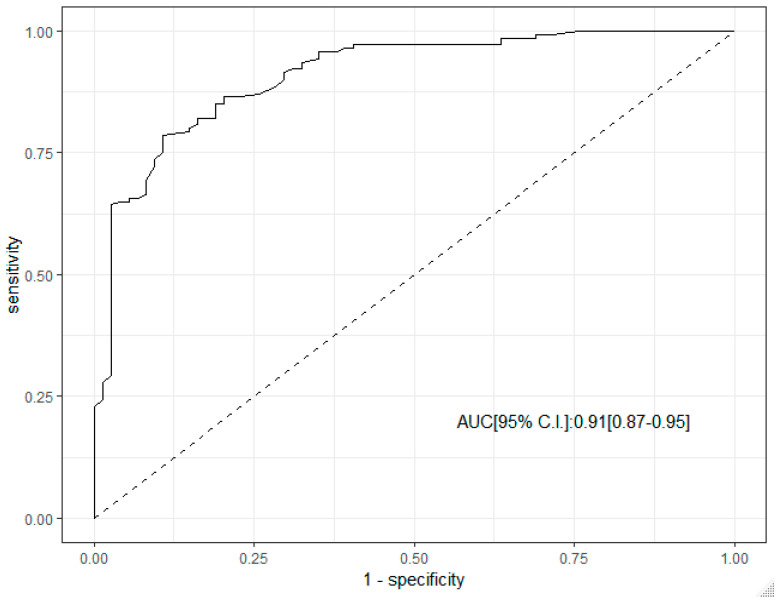
ROC curve analysis of SR in the diagnosis of malignant lymph node.

ROC curve analysis supported the predictive role of SR in the diagnosis of malignant LN. The corresponding AUC was 0.91 (95% C.I. 0.87–0.95), Figure 2.

Sensitivities and specificities of SR for the identification of benign or malignant lesions and for each malignant diagnosis according to the “best” cut-off point are reported in Table 2.

**Figure 3:**
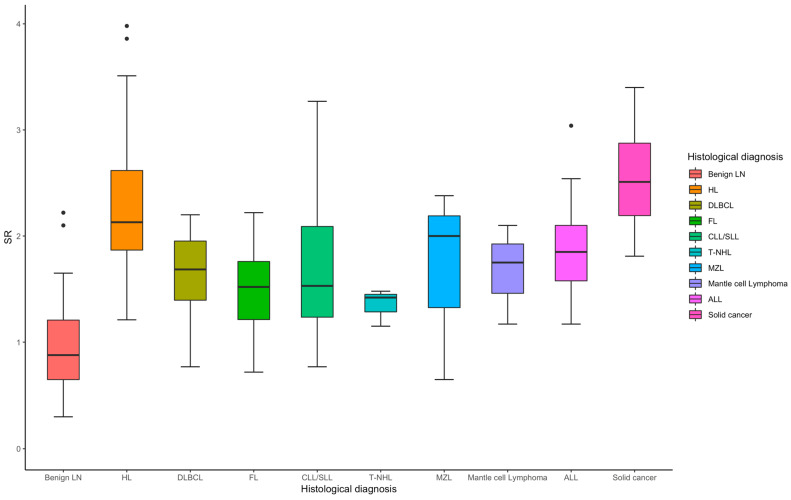
Box plot of SR in the diagnosis of benign and different subtypes of malignant lymph node.

For hematological neoplasms (*n* = 122), the median SR is 1.84, with sensitivity ranging (Table 2) from 0.71 to 0.85 and specificity from 0.81 to 0.96, Figure 3. The overall AUC is 0.91, indicating a strong diagnostic performance. Hodgkin Lymphoma (*n* = 48) stands out with exceptional sensitivity (0.94) and specificity (0.90), yielding an AUC of 0.97, reflecting excellent diagnostic accuracy. For non-hematological neoplasms (*n* = 18), the median SR is higher at 2.51 (Figure 3), with sensitivity ranging from 0.67 to 1.00, specificity between 0.82 and 0.96, and an AUC of 0.93, indicating strong diagnostic performance overall.

To assess the role of SR as an independent predictor of diagnosis, a set of multivariable logistic regression models including LN shape, echogenicity, hilum features, long axis diameter, vascularization, and ES was constructed and reported in Table 3. SR included in these base models was a significant independent predictor, both as a continuous and dichotomous variable (using the “best” cut-off value) of malignant status. The gain in predictive accuracy was 14% when SR was used as a continuous variable and 12% when SR was considered as a dichotomous variable using 1.4 as the optimal threshold [sensitivity 0.79 (0.72–0.85) and specificity 0.89 (0.82–0.96).

## 4. Discussion

In this study, we investigated the diagnostic accuracy of US-E in differentiating between malignant and benign superficial LN in patients suspected of lymphoma and explored its potential clinical utility as a non-invasive diagnostic tool.

Previous studies have shown that conventional US has limitations in differentiating benign from malignant LNs, particularly in cases with ambiguous characteristics such as mildly enlarged nodes or indeterminate vascular patterns [2,13]. In our study, although B-mode US features like shape, size, and echogenicity provided some predictive value, they were insufficient to reliably classify LNs nature. Indeed, the sensitivity of long axis size (≥1.5 cm) alone was only 0.66, and the specificity of shape (round vs. oval) was 0.73. In contrast to the findings of Toriyabe et al. [33] and Lyshchik et al. [25], who reported that 68% and 82% of benign lymph nodes (LNs) were oval, respectively, our study observed that only 42% of benign LNs had this shape. Furthermore, they also reported [25,33] that 81% and 75% of malignant LNs were round, our study found that 85% of malignant LNs were round. These differences highlight variations in the shape distribution of benign and malignant LN across studies, yet still underscore its potential role, even though these features lack high sensitivity and specificity.

In terms of echogenicity, our study found that hypoechoic LNs were predominantly associated with malignancy (sensitivity = 0.71, specificity = 0.68), which aligns with previous findings [33,34].

The absence of visible hilum due to replacement or effacement is considered an important criterion for malignant LNs and has been reported in 76–96% of malignant nodes [25,31,35,36,37].

Consistent with previous studies by Kanagaraju et al. [36], who reported that all 17 malignant lymph nodes (LNs) lacked a visible hilum (resulting in 100% sensitivity), and by Abdelgawad et al. [32], who observed hilum loss in 100% of malignant nodes compared to 27% in benign ones, our study found that 95% of malignant LNs showed no visible hilum. However, we also noted that a significant proportion (81.4%) of benign LNs also lacked a visible hilum. This suggests that the absence of a hilum, while more common in malignant LNs, is not exclusive to malignancy and may not be a definitive diagnostic feature on its own. Interestingly, some studies, such as one by Vassallo et al. [38], have documented a hyperechoic hilum in up to 51.5% of metastatic nodes, further complicating the utility of hilum presence or absence in distinguishing between benign and malignant conditions.

One key finding from our study was the additional diagnostic value of power Doppler US in LN evaluating vascularization. As previously shown, abnormal vascularity in LNs (e.g., hypervascularization with a high resistive index) is strongly associated with malignancy [8,9,10,39]. In our study, hypervascularization showed a high specificity (0.77) for malignancy, reinforcing the relevance of Doppler evaluation. In comparison, Teng et al. [39] reported a sensitivity of 67%, specificity of 76%, and overall accuracy of 71% for Doppler US in differentiating benign and malignant LN. Similarly, Lyshchik et al. [25] found that Doppler US had higher specificity (99%) than sensitivity (47%) for malignancy, further supporting the utility of vascular patterns as a key feature in lymph node assessment. These findings reinforce the potential of power Doppler US as a valuable adjunct to conventional imaging techniques in the accurate diagnosis of LN pathology.

Interestingly, among the malignant diagnoses, LNs from solid cancers exhibited distinct sonographic features. In the 18 cases of solid cancer, absence of the hilum was observed in 100% (18/18), hypoechogenicity in 94% (17/18), irregular shape in 89% (16/18), and vascularization in 89% (16/18) of cases. These findings underscore that ultrasonographic characteristics can differ significantly depending on the underlying malignant diagnosis. In particular, solid tumors tend to display a typical pattern marked by hilum loss, low echogenicity, and increased vascularization, which may not be equally present in other malignancies such as lymphomas. This highlights the importance of considering the histological type when interpreting ultrasound findings in suspicious LNs.

However, when combined with SR, the diagnostic accuracy improved, highlighting the complementary nature of these two techniques. This is consistent with prior research suggesting that combining elastography with Doppler US improves diagnostic performance compared to using either modality alone [25,28].

Our findings reveal that SR, as a measure of tissue stiffness, was significantly higher in malignant LNs compared to benign ones. ROC curve analysis supports the predictive role of SR, with an impressive area under the curve (AUC) of 0.91, indicating strong diagnostic performance. The high sensitivity (0.79–0.94) and specificity (0.80–0.96) observed for SR in detecting malignancy mirror those found in similar studies on US-E in other organ systems. For example, in breast and thyroid cancer, SR has been shown to correlate with malignancy, with malignant lesions generally exhibiting higher stiffness than benign ones [15,16]. Our study extends these findings to superficial LN, confirming that SR can be a reliable predictor of malignancy across a range of lymphoid and non-lymphoid neoplasms. Specifically, the performance of SR in distinguishing between hematologic malignancies, such as HL and DLBCL, was particularly notable. For HL (AUC = 0.97), SR exhibited exceptional diagnostic sensitivity (0.94) and specificity (0.90), supporting the hypothesis that lymphomas tend to be stiffer than benign reactive LNs due to the proliferation of malignant cells and fibrotic changes in the LN capsule and surrounding tissue. Our results align with previous studies that have demonstrated the ability of US-E to enhance the accuracy of LN evaluation, particularly in distinguishing malignancies, such as lymphoma and metastases, from benign conditions [21,22,23,27,28,29,30,31].

The independent role of SR as a predictor of malignancy was further confirmed by multivariable logistic regression, which demonstrated that when included in these models, SR added significant diagnostic value, enhancing the predictive accuracy of conventional US features such as shape, size, and vascularity.

These findings are in line with the working hypothesis that combining US-E with traditional sonographic features improves diagnostic precision, particularly when assessing complex lymphoid structures that exhibit overlapping characteristics in benign and malignant cases. The gain in predictive accuracy (14% for continuous SR, 12% for dichotomous SR) highlights the utility of SR as a non-invasive biomarker that can supplement standard sonographic evaluations and potentially guide the selection of targets for biopsy, improving diagnostic precision, particularly in cases with equivocal B-mode US findings.

While our study provides valuable insights into the diagnostic utility of ultrasound elastography in differentiating between benign and malignant superficial lymph nodes, several limitations should be acknowledged. Firstly, our study was conducted at a single center, which may limit the applicability of our findings to other institutions with different patient populations, equipment, device-specific settings, clinical practices, and operator experience, thereby potentially affecting external validity. We acknowledge this limitation and recognize the importance of expanding data collection across multiple centers to strengthen the generalizability of our results. Furthermore, we will consider the use of publicly available imaging datasets in future research to enable independent validation and enhance the robustness of our findings.

Additionally, while the total number of patients was relatively large, the diversity of cases (types of lymphoma and metastases) may not fully represent all disease variants. The stratification into smaller groups of specific neoplasms may reduce the robustness of conclusions for less common pathologies. Furthermore, the enrollment criteria for our study were based on clinical suspicion of lymphoma and indication for nodal biopsy. This selection criterion may not accurately reflect the general population of patients with enlarged LNs as those with viral or bacterial infections were excluded and may introduce selection bias and affect the representativeness of our study population. However, in patients with multiple lymphadenopathies, only the LN that was deemed most representative or suspicious for malignancy was included in the analysis. This may limit the generalizability of some findings, particularly in diseases such as lymphoma, where nodal involvement may be more diffuse and variable. Future studies could benefit from a more granular analysis across multiple nodes within the same patient to better capture this heterogeneity.

Additionally, the interpretation of US and US-E images relied on the expertise of the operators and on the quality of the equipment, which may introduce variability and bias into the results, despite efforts to standardize imaging protocols and training. Moreover, despite efforts to reach agreement in cases of discrepancy between operators, interobserver variability in image interpretation and ES evaluation may still exist and impact the consistency of our results (inter-observer and inter-equipment variability).

The inter-rater reliability for SR measurement was excellent, with an intraclass correlation coefficient (ICC) of 0.88 (95% CI: 0.82–0.94). However, a key limitation is that ICCs were calculated based on the independent review of identical, pre-acquired elastographic cineloops by both operators, therefore reflecting only interpretation reproducibility and not accounting for potential variability introduced during image acquisition.

Lastly, the use of SR as the main parameter for diagnosis may not be universally applicable, as it depends on factors such as LN location, depth, and proximity to surrounding structures that could interfere with accurate readings. Furthermore, it is important to note that SR values and their corresponding thresholds are likely to be platform-dependent, as different US-E systems may vary in their calibration, processing algorithms, and measurement techniques. Therefore, our findings may not be directly generalizable across all elastography platforms without appropriate calibration or standardization procedures, which should be taken into account when interpreting or applying these results in other clinical settings.

Despite these limitations, our comprehensive approach with the inclusion of both conventional B-mode US and power Doppler alongside US-E provides a multi-dimensional assessment of LN characteristics. This method enables a more nuanced understanding of lymph node pathology compared to traditional imaging techniques.

RTE shows promise in assessing therapeutic response in HL, particularly in patients receiving brentuximab vedotin. Squillaci et al. [40] demonstrated that elastographic parameters correlate with clinical and pathological responses to treatment, suggesting that US-E can detect treatment effects early. Similarly, Kovaleva et al. [41] successfully used US-E for the intermediate evaluation of therapeutic response, highlighting its potential to monitor tumor shrinkage and fibrosis, in an HL setting. These findings support the integration of elastography into clinical practice for non-invasive, real-time monitoring of therapy response. As elastographic technology advances, its role in lymphoma treatment monitoring is likely to expand, offering valuable insights and reducing the need for invasive procedures. Moreover, further investigation into the role of US-E in monitoring treatment response in patients with lymphoma and other malignancies is warranted. US-E could potentially be used to assess changes in LN stiffness over time, offering a non-invasive method for evaluating therapeutic efficacy and detecting relapse.

Recent studies highlight the growing potential of artificial intelligence (AI) in improving diagnostic accuracy in elastographic imaging, particularly for LN malignancies. For instance, NeuralSeg, an AI-based algorithm, showed high specificity and AUC in predicting mediastinal LN malignancy from elastography images, using predefined cutoffs and color stiffness thresholds [42]. In breast imaging, integrating SWE significantly boosted diagnostic performance, with expert radiologists achieving an AUC of 0.934, outperforming the AI model. While the AI showed lower accuracy, its consistent performance across test data suggests strong robustness and generalizability [43]. Currently, AI and radiomics serve as valuable complements to expert clinical judgment rather than replacements. However, as datasets grow and models advance, AI’s role in the US-E is expected to expand, potentially enhancing diagnostic precision and supporting broader clinical adoption.

## 5. Conclusions

This study highlights the value of US-E as a complementary tool to conventional and power Doppler US for the non-invasive assessment of LNs in patients suspected of lymphoma. While traditional B-mode US features like shape, size, and echogenicity provide some insights, they alone are insufficient for reliable differentiation between benign and malignant LNs. The SR emerged as a robust indicator of malignancy, particularly in hematologic conditions such as Hodgkin lymphoma, demonstrating high sensitivity and specificity. Combining US-E with B-mode and Doppler ultrasound enhances diagnostic accuracy. Although further validation in larger and more varied patient cohorts is necessary, US-E shows promise as a reliable, non-invasive approach for LN evaluation and monitoring treatment response in lymphoma patients.

Importantly, US-based assessments such as US-E play a pivotal role in supporting shared decision-making and patient counseling, especially for individuals hesitant to undergo biopsy. By providing a non-invasive, real-time evaluation of lymph node characteristics and stiffness, these tools offer a risk-informed alternative that can guide patient-centered discussions.

## Figures and Tables

**Table 1:** Results according to US criteria for benign and malignant lymph nodes.

Sonography Criteria		Lymph Nodes		Sensitivity (95%CI)	Specificity (95%CI)
		Benign (*n* = 74)	Malignant (*n* = 140)		
**B-mode**					
Size of long axis diameter *	<1.5 cm	7	11	0.39 (0.17–0.64)	0.66 (0.59–0.72)
	≥1.5 cm	67	129		
Shape	Slender	31	21	0.60 (0.45–0.73)	0.73 (0.66–0.80)
	Round	43	119		
Hilum	Present	14	7	0.67 (0.43–0.85)	0.69 (0.62–0.75)
	Absent	60	133		
Echogenicity	Hypertrophic cortical	12	6	0.67 (0.41–0.87)	0.68 (0.61–0.75)
	Hypo	62	134		
Vascularization +	Normal vascularization	36	16	0.69 (0.55–0.81)	0.77 (0.69–0.83)
	Hypervascularization	38	124		
**Elastography**					
Pattern ES	1	1	1	0.47 (0.38–0.57)	0.82 (0.73–0.89)
	2	23	10		
	3	33	52		
	4	17	77		
Strain ratio, median (range)		0.88 (0.3–2.22)	1.84 (0.65–3.98)	0.89 (0.82–0.96)	0.78 (0.71–0.86)

* long axis greater than or equal to 1.5 cm; + intranodal arterial vessels with resistive index value ≥ 1.6. ES according to the four pattern system, scores 1 = soft LN that is predominantly red, with <10% of the area colored as blue); score 2: moderately soft LN that is predominantly red and green, with 10–50% of the area shown as blue; score 3: moderately stiff LN that is predominantly blue and green, with 50–90% of the area shown as blue; score 4: stiff LN that is predominantly >90% blue.

**Table 2:** Accuracy of SR for malignant findings.

Histologic Diagnosis	SR Median (Range)	Sensitivity	Specificity	AUC (95% CI)	Threshold
Hematological neoplasms (*n* = 122)	1.84 (0.65–3.98)	0.79 (0.71–0.85)	0.89 (0.81–0.96)	0.91 (0.87–0.95)	1.40
Hodgkin lymphoma (*n* = 48)	2.13 (1.21–3.98)	0.94 (0.85–1)	0.90 (0.94–0.96)	0.97 (0.94–0.99)	1.50
Diffuse large B cell lymphoma (*n* = 28)	1.68 (0.77–2.2)	0.86 (0.71–0.96)	0.8 (0.70–0.88)	0.89 (0.82–0.95)	1.26
Follicular lymphoma (*n* = 22)	1.52 (0.72–2.22)	0.91 (0.77–1)	0.65 (0.54–0.76)	0.84 (0.76–0.93)	1.06
CLL/SLL (*n* = 11)	1.51 (0.77–3.27)	0.9 (0.7–1)	0.65 (0.54–0.74)	0.82 (0.69–0.96)	1.05
T cells NHL (*n* = 3)	1.42 (1.15–1.48)	1 (1–1)	0.69 (0.58–0.8)	0.83 (0.69–0.98)	1.15
Marginal zone lymphoma (*n* = 3)	2 (0.65–2.38)	0.97 (0.82–1)	0.67 (0–1)	0.74 (0.27–1)	1.82
Mantle cell lymphoma (*n* = 3)	1.75 (1.17–2.1)	1 (1–1)	0.78 (0.6–0.81)	0.88 (0.7–1)	1.16
Acute lymphoblastic leukemia (*n* = 4)	1.85 (1.17–3.04)	0.97 (0.93–1)	1 (1–1)	0.99 (0.97–1)	1.73
Non hematological neoplasms (*n* = 18)	2.51 (1.81–3.04)	0.83 (0.67–1)	0.9 (0.82–0.96)	0.93 (0.88–0.99)	1.49

**Table 3:** A Multivariable logistic regression model including size of long axis diameter, shape, hilum, echogenicity, vascularization, and ES for prediction of malignant diagnosis.

Characteristic	Continuous SR		SR < 1.4 SR ≥ 1.4	
	OR [95% CI]	*p*-Value	OR [95% CI]	*p*-Value
Size of long axis diameter *	0.92 [0.16–5.21]	0.92	1.11 [0.26–4.77]	0.91
Shape	0.76 [0.25–2.15]	0.61	0.99 [0.39–2.49]	0.98
Hilum	1.06 [0.45–8.63]	0.94	1.77 [0.44–8.33]	0.41
Echogenicity	0.68 [0.16–3.13]	0.61	0.92 [0.26–3.61]	0.88
Vascularization ^+^	2.74 [1.07–7.17]	0.04	3.92 [1.58–10.14]	0.004
ES	0.89 [0.35–2.22]	0.81	1.58 [0.65–3.79]	0.30
SR, continuous	58.8 [1.77–251]	<0.0001		
SR, dichotomous (SR < 1.4; SR ≥ 1.4)			21.4 [8.9–57.1]	<0.0001
AUC [95% CI]	0.92 [0.88–0.96]		0.89 [0.84–0.94]	
Gain in predictive accuracy; % (*p*-value)	14% (<0.0001)		12% (<0.0001)	

* long axis greater than or equal to 1.5 cm; + intranodal arterial vessels with resistive index value ≥ 1.6.

## Data Availability

The data presented in this study are available on request from the corresponding author due to (specify the reason for the restriction).

## Abbreviations

The following abbreviations are used in this manuscript:

LN	Lymph Node
US	Ultrasound
US-E	Ultrasound Elastography
SE	Strain Elastography
SWE	Shear Wave Elastography
ARFI	Acoustic Radiation Force Impulse
TE	Transient Elastography
SR	Strain Ratio
ES	Elastography Score
ROI	Region of Interest
RTE	Real-time Tissue Elastography
AUC	Area Under the Curve
ROC	Receiver Operating Characteristic
CI	Confidence Interval
HL	Hodgkin Lymphoma
DLBCL	Diffuse Large B Cell Lymphoma
FL	Follicular Lymphoma
CLL/SLL	Chronic Lymphocytic Leukemia/Small Lymphocytic Lymphoma
T-NHL	T-cell Non-Hodgkin Lymphoma
MZL	Marginal Zone Lymphoma
NHL	Non-Hodgkin Lymphoma
ALL	Acute Lymphoblastic Lymphoma
